# A Comprehensive and Comparative Analysis of the Fucoidan Compositional Data Across the Phaeophyceae

**DOI:** 10.3389/fpls.2020.556312

**Published:** 2020-11-25

**Authors:** Nora M. A. Ponce, Carlos A. Stortz

**Affiliations:** Departamento de Química Orgánica, Ciudad Universitaria, Facultad de Ciencias Exactas y Naturales, Consejo Nacional de Investigaciones Científicas y Técnicas, Centro de Investigaciones en Hidratos de Carbono (CIHIDECAR/CONICET), Universidad de Buenos Aires, Buenos Aires, Argentina

**Keywords:** fucoidans, brown seaweeds, phaeophyceae, taxonomy, phylogeny

## Abstract

In the current review, compositional data on fucoidans extracted from more than hundred different species were surveyed through the available literature. The analysis of crude extracts, purified extracts or carefully isolated fractions is included in tabular form, discriminating the seaweed source by its taxonomical order (and sometimes the family). This survey was able to encounter some similarities between the different species, as well as some differences. Fractions which were obtained through anion-exchange chromatography or cationic detergent precipitation showed the best separation patterns: the fractions with low charge correspond mostly to highly heterogeneous fucoidans, containing (besides fucose) other monosaccharides like xylose, galactose, mannose, rhamnose, and glucuronic acid, and contain low-sulfate/high uronic acid proportions, whereas those with higher total charge usually contain mainly fucose, accompanied with variable proportions of galactose, are highly sulfated and show almost no uronic acids. The latter fractions are usually the most biologically active. Fractions containing intermediate proportions of both polysaccharides appear at middle ionic strengths. This pattern is common for all the orders of brown seaweeds, and most differences appear from the seaweed source (habitat, season), and from the diverse extraction, purification, and analytitcal methods. The Dictyotales appear to be the most atypical order, as usually large proportions of mannose and uronic acids appear, and thus they obscure the differences between the fractions with different charge. Within the family Alariaceae (order Laminariales), the presence of sulfated galactofucans with high galactose content (almost equal to that of fucose) is especially noteworthy.

## Introduction: Aim of the Review

Fucoidans are sulfated polysaccharides present in the cell walls of the Phaeophyceae (brown seaweeds) composed usually by fucose (Fuc) as the main monosaccharide, but accompanied by very variable amounts of other monosaccharides like galactose (Gal), xylose (Xyl), mannose (Man), rhamnose (Rha), and/or glucuronic acid (GlcA). The scientific literature on different aspects of fucoidans is steadily growing, mostly due to the diverse biological activities found for samples from many different species of seaweeds. This bioactivity (antiviral, anticoagulant, antitumoral, antioxidant, among others) has been reviewed extensively (Cosenza et al., 2017; Senthilkumar et al., 2017; Wang et al., 2019). Many studies attempted to explore the structural details of fucoidans, but it was very difficult to find a common trait in the different fucoidans so far analyzed (Bilan and Usov, 2008; Kopplin et al., 2018). This marks a big difference with red seaweed sulfated galactans, showing an unchallenged disaccharidic repeating structure modified by the position of sulfation, the series of the α-galactose units and its possible presence as a 3,6-anhydro ether (Usov, 2011). For these galactans, it has been found that the taxonomic order (or sometimes the family) to which the seaweed yielding the galactan belongs has a strong influence on the characteristics of these galactans, i.e., chemotaxonomy appears to be in effect (Miller, 1997; Stortz and Cerezo, 2000). For instance, within the brown seaweeds, it has been postulated that the fucoidans from the Laminariales tend to have just α-3-linked Fuc units, whereas those of the Fucales show more proportions of a α-(1,3)-α-(1,4) alternating structure (Deniaud-Bouët et al., 2014), as a chemotaxonomical trait related to structure. A previous review by Ale et al. (2011) has tried to establish some relationship with taxonomy, with the focus set on extraction methods, qualitative compositional data, and structural features. In this review, compositional data on fucoidans originated in different taxonomic groups of the Phaeophyceae will be presented. Two hypotheses are put into consideration: (a) that there is a relationship between some of these compositional features and the taxonomic classification, and (b) that various other factors produce the differences in composition.

## Taxonomy of the Phaeophyceae

**FIGURE 1:**
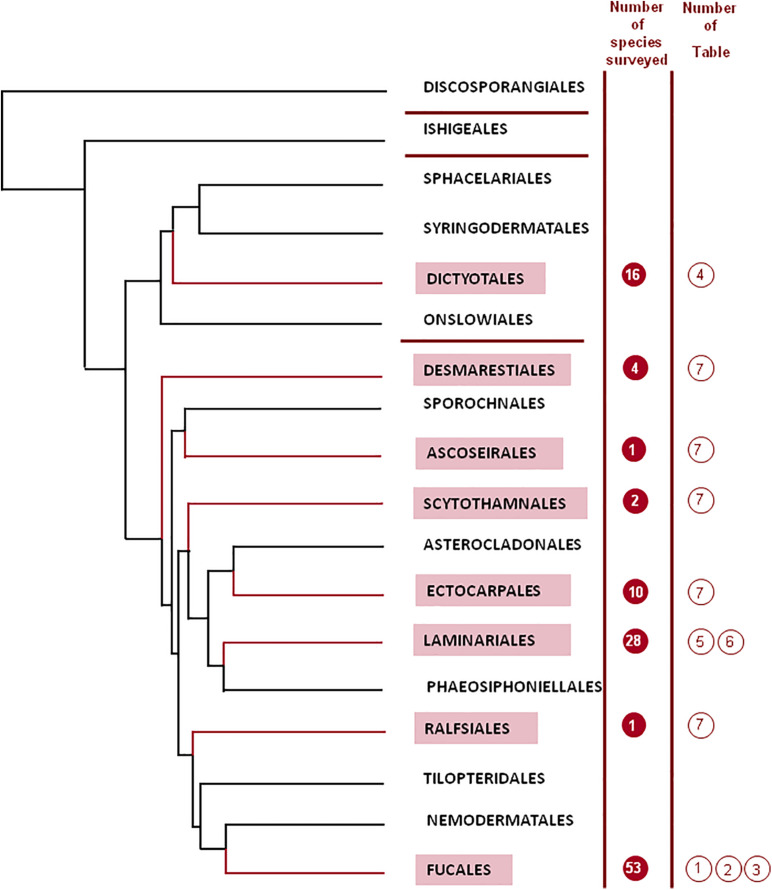
Phylogenetic tree for the different orders of the Phaeophyceae (adapted from Silberfeld et al., 2014; reproduced with kind permission from the authors). One diverging branch from the order Scytothamnales containing the family Bachelotiaceae has been removed from the figure for the sake of simplicity.

The taxonomy of brown algae (Heterokonta, Ochrophyta, Phaeophyceae) had many controversies throughout the history (Silberfeld et al., 2014). Order delineation in the Phaeophyceae has traditionally been based on the type of life cycle, reproductive aspects, mode of growth, and filamentous vs. parenchymatous construction of the thallus (Rousseau and de Reviers, 1999a, b). However, with the advent of molecular systematics, new insights were brought, thoroughly reshaping the evolutionary concepts of brown algae. Rousseau and de Reviers (1999b) and de Reviers et al. (2007) have provided a detailed evolution of classificatory concepts within the Phaeophyceae. Several changes in the classification at the ordinal level have been set between the Oltmanns (1922), comprising 8 orders to the present times classification, encompassing 18 orders (Silberfeld et al., 2014; Figure 1). Major changes were produced after the DNA sequencing of brown seaweeds started in 1993 (Draisma et al., 2003; de Reviers et al., 2007). Different molecular markers can be used, but phylogenetic studies of Phaeophyceae have mostly utilized the rDNA sequences, which include four subunits (18S, 5.8S, 26S, and 5S), containing regions which are highly conserved as well as others highly variable. Most information arose from studies on the 18S subunit of rDNA, although those studies had limited results for more recent Phaeophycean lineages (Tan and Druehl, 1996). In this way, Rousseau et al. (2001) utilized the 26S sequence, which altogether with a larger taxonomic sampling, solved some of the earlier divergences. Thus, a phylogenetic tree was constructed (Draisma et al., 2001, 2003). It has been concluded that morphological characters, many times useful to understand the ecology of brown seaweeds, have no value at all for phylogeny. Different degrees of organization, diffuse or apical growth, or life stages have appeared and disappeared repeatedly in the history of the different taxonomic groups.

Silberfeld et al. (2014) have introduced a thorough phylogenetic analysis based on a dataset generated previously (Silberfeld et al., 2011), including seven markers, for a total of 6804 nucleotides, determined for 91 Phaeophycean taxa, including minor orders for which there were very few studies. In this way, the shape of phylogenetic trees changed sharply the previous knowledge (Silberfeld et al., 2011; Charrier et al., 2012). Figure 1 depicts the outcome of the tree for the 18 orders determined by Silberfeld et al. (2014), grouped in four subclasses (Discosporangiophycidae and Ishigeophycidae, including one order each, Dictyotophycidae, including four orders, and Fucophycidae, including the remaining 12 orders).

## Polysaccharides From the Phaeophyceae: The Fucoidans

Most macroalgae exhibit polysaccharides as their most abundant constituents. Taking into account their function, they can be classified into two main groups: storage and structural polysaccharides. The formers are polymers such as starch/glycogen or laminaran considered as food reserve materials, whereas the latters are structural elements of the cell walls, intercellular tissues and mucilaginous matrix. Sulfated polysaccharides are a group of anionic structural polysaccharides, useful for the seaweed in the marine environment to avoid desiccation. Their gross composition is characteristic of each algal group (galactans in red seaweeds, fucoidans in brown seaweeds, rhamnoglucuronans, and arabinogalactans in green seaweeds, van den Hoek et al., 1996), whereas more or less subtle differences appear often depending on the order, family, genus and species, as well as sometimes on the season, geographic location, or reproductive stage (Mackie and Preston, 1974). Other roles of the polysaccharides might include participations in cell-cell communication (Deniaud-Bouët et al., 2014), and in cell division processes (Skriptsova, 2015).

In macroalgae, the cell walls comprise a fibrillar skeleton immersed in an amorphous matrix. In the case of the Phaeophyceae, the fibrillar skeleton is mainly made up of cellulose [a linear β-(1→4)-glucan], and the surrounding matrix is composed predominantly by alginic acid or its salts, together with a system of sulfated polysaccharides (the fucoidans; Mackie and Preston, 1974). In this way, the cell wall is composed of two different layers: the inner layer consisting of a skeleton of microfibrils providing rigidity to the cell wall, and the outermost layer, which is usually observed as a poorly crystalline matrix in which the set of microfibrils is embedded. There is also evidence that the matrix does not penetrate the fibers, but remains attached to this layer through hydrogen bonds (Davis et al., 2003). It has been suggested that fucoidans might play a key role in cell wall architecture, cross-linking cellulose and alginates (Kloareg et al., 1986). Besides this function, as occurs with other sulfated polysaccharides, the fucoidans help to protect the plant from desiccation. When the fronds are in contact with sea water the sulfate hemiester groups are strongly associated with magnesium ions, which are highly hydrated and thus retain water in the fronds (Percival, 1979). In a more modern model for the Fucales (Deniaud-Bouët et al., 2014, 2017; Torode et al., 2016), it has been proposed that two networks are assembled in the cell wall; the first one contains the fucoidans interlocking a cellulose (or other β-glucans) network, and the second one contains alginate crosslinked by polyphenols. The rigidity is controlled by the alginate structure and its calcium cross-linking capabilities, whereas the fucoidans participate mostly in adaptation to the osmotic stress.

More than one century ago, Kylin has isolated for the first time (from different seaweed species of the genera *Fucus*, *Laminaria*, and *Ascophyllum*) a group of sulfated polysaccharides with a high Fuc content and called them “fucoidin” (Kylin, 1913). Originally the name fucoidin (later changed to the more systematic fucoidan) was coined for the polysaccharides from those species, but this term was rapidly extended to any fucose-rich polysaccharides, including not only those becoming from brown seaweeds, but also to those present in echinoderms (Olatunji, 2020). As noted above, fucoidans are sulfated polysaccharides present mainly in the intercellular tissue of mucilaginous matrix of the cell walls of brown algae (Deniaud-Bouët et al., 2017).

**FIGURE 2:**
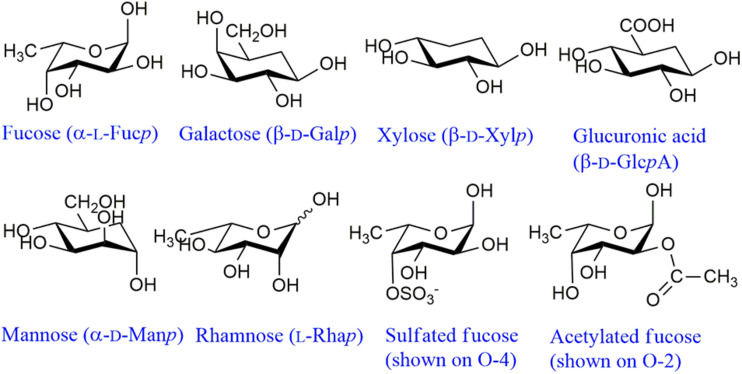
Main structural monosaccharidic units of fucoidans. These monosaccharides can appear as terminal non-reducing units or linked through any of the free hydroxyl groups. Usually Fuc and GlcA appear linked through O-3 or O-4, Xyl through O-4, Gal through O-3 or O-6 and Man through O-2 (Sakai et al., 2003; Bilan et al., 2010, 2017, 2018). The structural features of Rha are unknown. For representative structures of fucoidans (see Deniaud-Bouët et al., 2017).

Fucoidans comprise a family of diverse molecules containing, in addition to Fuc, varying proportions of Gal, Man, Xyl and GlcA (Figure 2). Acetate esters have also been found, especially in modern studies (see below). In the early studies extensive purification was carried out in an effort to isolate a “fucan” containing only Fuc residues, assuming that the remaining monosaccharides were originated in other, contaminating polysaccharides. Nevertheless, even in the allegedly pure samples, small proportions of Gal, Xyl, and/or uronic acid persisted (Percival, 1979). Later, only in a few species a pure fucan was isolated after purification (see below). Thus, most of the samples so far isolated are heterofucans (Deniaud-Bouët et al., 2014).

## Fucoidans From Different Species of Phaeophyceae

In this section, the main chemical characteristics of fucoidans extracted from different species of brown seaweeds reported so far to the best of our knowledge (with compositional data provided) will be described in tabular form. They will be shown separately for each of the different orders (Figure 1). When numerous species of an order were studied, separations in families or genera are also displayed. It is worth noting that depending on the way that the analyses were expressed in the original papers, the uronic acids in the following tables were indicated as a percentage of the total sample (in most cases) or as part of the molar ratio of all the monosaccharides. Thus, these molar ratios might or might not include the uronic acid components. The main monosaccharidic units appearing in fucoidans are shown in Figure 2. When the authors have isolated a large number of fractions, only those more abundant or representative are listed in the tables. The reported presence of acetyl groups is indicated qualitatively with the “Ac” acronym. It should be noted that the geographic location and season of harvest of the seaweed can also have significant effects on the composition of the extracted fucoidans (e.g., Zvyagintseva et al., 2003). The extraction and fractionation procedures are schematically displayed, neglecting defatting and depigmenting steps, as well as usual procedures like dialysis or single alcohol precipitations. The methods used for monosaccharide and sulfate quantitation are also shown.

### Fucales

As expected, samples of fucoidans from this is order were the most studied. Samples from five different families of the Fucales have been studied. Two species from the Fucaceae, i.e., *Fucus vesiculosus* and *Ascophyllum nodosum* appear in the earlier studies by Kylin (1913). The polysaccharides from these species were studied extensively by different research groups (see below). However, the family with more species studied was the Sargassaceae. Considering only the genus *Sargassum*, studies on the fucoidans from 26 different species were found in the current survey.

The extraction of fucoidans from *Fucus vesiculosus* was originated in the early Kylin studies, when Fuc was characterized after hydrolysis as phenyl-L-fucosazone; pentoses in the hydrolyzate were also reported (Kylin, 1913). Different products from this species were extensively studied (Table 1). Originally, the presence of Xyl was ascribed to a contaminating xylan that accompanied the fucoidan (Percival and McDowell, 1967). As a matter of fact, they reported the isolation of a xylan, although uronic acid residues were found in the xylan fraction and, furthermore, the authors were not able to separate any fraction composed just by Fuc residues. The studies by Nishino et al. (1994a) on a commercial sample from this seaweed were highly comprehensive: they were able to separate 13 different fractions and analyze them thoroughly, showing structures ranging from typical fucans (containing mainly Fuc and sulfate, and free of uronic acids) to heteropolysaccharides with low sulfate content and high content of uronic acids. In a minor fraction, they were able to find an appreciable amount of glucosamine (11.5%). In an interesting study using microwave extraction of this seaweed, Rodríguez-Jasso et al. (2011) showed that depending on the pressure and extraction time, fucoidans with different ratios Fuc/Gal were obtained (ranging from 100% Fuc to a 1:1 ratio), plus variable proportions of Xyl and sulfation degrees. Another species from the same genus that has been studied is *Fucus evanescens*. Zvyagintseva et al. (1999) separated the polysaccharides using a chromatography system on a hydrophobic resin. It is interesting to note that in a subsequent work Zvyagintseva et al. (2003) analyzed specimens of three different seaweeds (*F. evanescens*, *Laminaria cichorioides*, and *Saccharina japonica*) collected at different places, at various stages of development and at different seasons, and found some notable differences, particularly for the *F. evanescens* equivalent fractions obtained in different geographic locations (ratio Fuc/sulfate between 1 and 2.1; Fuc proportion from 56 to 80%; molecular masses from 14–40 to 150–500 kDa).

**TABLE 1:** Reported compositions of the fucoidans from the family Fucaceae (Fucales).

Species	Extraction	Purification/	Acronym	Monosaccharide composition (moles %)									Sulfate		UA (%)	References
		Fractionation^a^		Method^b^	Fuc	Xyl	Gal	Man	Glc	Rha	GlcA	Others	Method^c^	%		
*Fucus vesiculosus*	HCl pH 2	Ethanol ppt	F1	GC	50	15	4	17	14				Pb	4	22	Medcalf and Larsen (1977a)
	HCl pH 2	Ethanol ppt	F2	GC	70	7	8	4	11				Pb	25	6	“
	HCl 0.01M+CaCl_2_ 1%			GC	79	10	6	3	2				Tit	31	14	Mabeau and Kloareg (1987)
	pH 7.5+CaCl_2_ 1%	EtOH+TCA 10%	FF	GC	84	2	13		1				Tit	26	4	Mabeau et al. (1990)
	Triton 0.5%, pH 7.5+CaCl_2_ 1%	EtOH+TCA 10%	TF	GC	60	10	14	10	6				Tit	14	9	“
	HCl 0.01M+CaCl_2_ 1%		HT	GC	87	4	5	2	2				Tit	39	17	“
	Na_2_CO_3_ 3%	HCl 0.01M ppt	OHF	GC	78	11	5	3	3				Tit	30	9	“
			SigmaTM	GC	92	4	3	2					DP	23	8	Nishino et al. (1994a)
	SigmaTM	SEC+AEC	I_1.8_	GC	90	3	5	2					DP	32	3	“
	SigmaTM	SEC+AEC	II_1.35_	GC	94	1	5	tr.					DP	33	–	“
	SigmaTM	SEC+AEC	II_2_	GC	94	1	5						DP	36	–	“
	SigmaTM	SEC+AEC	III1_1.5_	GC	93	2	5						DP	34	–	“
	H_2_O, r.t.		F1	GC	55	11	9		25				DP	6	39	Rupérez et al. (2002)
	HCl 0.1M		F3	GC	89	6	5						DP	11	9	“
	CaCl_2_ 2% hot	PQA		GC	67	6	13	8	6				DP	24	10	Cumashi et al. (2007)
	CaCl_2_ 2% hot			GC	59	13	10	3	14				EA	18	7	Bittkau et al. (2020)
	CaCl_2_ 2% hot	PQA		HPLC	83	6	7	3	1				DP	25	1	Zhang et al. (2015)
*Fucus ceranoides*	HCl 0.01M+CaCl_2_ 1%			GC	80	10	7	4					Tit	31	12	Mabeau and Kloareg (1987)
*Fucus distichus*	CaCl_2_ 2% hot	PQA + AEC	F_1_	GC	84	10	3	2	1				DP	24	–	Bilan et al. (2004)
	CaCl_2_ 2% hot	PQA + AEC	F_3_	GC	83	9	4	2	1				DP	24	–	“
	CaCl_2_ 2% hot	PQA + AEC	F_4_	GC	96	2	2					Ac	DP	35	–	“
*Fucus evanescens*	HCl 0.4% r.t.	HC	F-1	HPLC	90	3	1		6				DP	∼12	ND	Zvyagintseva et al. (1999)
	HCl 0.4% r.t +H_2_O hot	HC	F-2	HPLC	91	7			1				DP	∼25	ND	“
	CaCl_2_ 2% hot	PQA + AEC	F_3_	GC	67	16	9	7					DP	29	11	Bilan et al. (2002)
	CaCl_2_ 2% hot	PQA + AEC	F_4_	GC	94	3	3					Ac	DP	46	–	“
	HCl pH 2-2.3 hot	AEC	FeF	HPLC	87	2	2	4	1				DP	28	ND	Anastyuk et al. (2012b)
	HCl 0.2M hot		Sterile	HPLC	69	7	9	8	6	1				ND	ND	Skriptsova et al. (2012)
	HCl 0.2M hot		Reprod.	HPLC	77	5	5	3	10					ND	ND	“
	HCl pH2-2.3		FeF	HPLC	78	8	10	4				Ac	DP	23	ND	Prokofjeva et al. (2013)
	CaCl_2_ 2% hot			GC	96		4						EA	27	4	Bittkau et al. (2020)
^d^	Enz.pH6 + CaCl_2_ 2%	AEC	FeF2	PAD	75	3	15	2	1	1		HexA 3	DP	35	^e^	Nguyen et al. (2020)
^d^	Enz.pH6 + CaCl_2_ 2%	AEC	FeF3	PAD	88	2	9					HexA 1	DP	39	^e^	“
*Fucus serratus*	HCl 0.01M+CaCl_2_ 1%			GC	76	18	5	1					Tit	22	15	Mabeau and Kloareg (1987)
	CaCl_2_ 2% hot	AEC	F_3_	GC	86	6	4	2	1			Ac	DP	22	–	Bilan et al. (2006)
	CaCl_2_ 2% hot	AEC	F_4_	GC	94	3	3					Ac	DP	32	–	“
	CaCl_2_ 2% hot	PQA + AEC		GC	69	7	13	6	5				DP	29	8	Cumashi et al. (2007)
	CaCl_2_ 2% hot			GC	41	10	4	2	43				EA	12	6	Bittkau et al. (2020)
*Fucus spiralis*	HCl 0.01M+CaCl_2_ 1%			GC	90	7	3	tr.					Tit	36	10	Mabeau and Kloareg (1987)
	CaCl_2_ 2% hot	PQA		GC	80	7	7	3	3				DP	26	8	Cumashi et al. (2007)
*Ascophyllum nodosum*	HCl 0.2M	AP/R	Ascoph.	CC	49	51							BC	12	19	Larsen et al. (1966)
	HCl 0.2M +AP/R	CaCl_2_ 0.04M+CE	F_2_	CC	86	14							BC	30	3	“
	H_2_O + OA pH 2.8^f^	CaCl_2_ 2%		GC	70	14					16		JL	21	11	Percival (1968)
	HCl pH 2	Ethanol ppt	F1	GC	37	29	3	21	11				M	13	26	Medcalf and Larsen (1977a)
	HCl pH 2	Ethanol ppt	F2	GC	73	11	2	10	5				M	21	16	“
	HCl pH 2	Ethanol ppt	F3	GC	81	9	2	4	4				M	25	6	“
	HCl pH 2	Ethanol ppt	F4	GC	34	14	27	15	10				M	15	7	“
	HCl pH 2	Ethanol ppt	F5	GC	71	7	14	4	4				M	8	7	“
	HCl pH 2	CaCl_2_ 1M+AP/R		GC	44	4	40	4				HexA 8	M	15	8	Medcalf et al. (1978)
	CaCl_2_ 2% hot	PQA		GC	67	11	12	7	3				DP	24	9	Cumashi et al. (2007)
	H_2_O + HCl 0.2M	AP/R		HPLC	47	40	2	10	1				DP	10	21	Nakayasu et al. (2009)
	H_2_O + HCl 0.2M	AP/R		HPLC	82	8	7	2	1				DP	24	2	Zhang et al. (2015)
	HCl 0.1M, MW^g^	CaCl_2_ 2%		PAD	40	14	6	11			24		DP	27	^e^	Yuan and Macquarrie (2015)
*Ascophyllum mackaii*	H_2_O hot	CaCl_2_ 1%+AP/R	AMF	HPLC	57	4	16	9	2	2	11		DP	22	^e^	Qu et al. (2014)
*Pelvetia canaliculata*	pH 7.5+CaCl_2_ 1%	EtOH+TCA 10%	FF	GC	82	4	10	2	2				Tit	29	4	Mabeau et al. (1990)
	Triton 0.5%, pH 7.5+CaCl_2_ 1%	EtOH+TCA 10%	TF	GC	65	13	11	6	5				Tit	20	6	“
	HCl 0.01M+CaCl_2_ 1%		HT	GC	81	9	7	2	1				Tit	40	2	“
	Na_2_CO_3_ 3%	HCl 0.01M ppt	OHT	GC	90	4	4	1	1				Tit	33	4	“
*Silvetia babingtonii*	HCl pH 2-2.3 hot	AEC	SbF	HPLC	77	5	12	6					DP	25	ND	Anastyuk et al. (2012b)
	HCl 0.2M hot		Sterile	HPLC	71	7	6	5	10					ND	ND	Skriptsova et al. (2012)
	HCl 0.2M hot		Reprod.	HPLC	80	6	6	4	4					ND	ND	“

*^a^Key: AEC, anion exchange chromatography; SEC, size-exclusion chromatography; HC, hydrophobic chromatography; CE, cation exchange; PQA, precipitation with quaternary ammonium salts; AP/R alcohol precipitation and redissolution.*

*^b^Key for the less common abbreviations: PAD, HPAEC with pulse amperometric detector; GC, gas chromatography; CC, column chromatography on carbon-Celite.*

*^c^Key: DP, method of Dodgson and Price (1962) or equivalent; Pb, titration with lead nitrate (Medcalf et al., 1972); EA, elemental analysis; Tit, titration with cetylpyridinium chloride, pH 1.5 (Scott, 1960); BC, method of barium chloranilate (Lloyd, 1959).*

*^d^Analyzed as Fucus distichus subsp. evanescens.*

*^e^The information for the uronic acid is included in the molar ratio of monosaccharides.*

*^f^Oxalic acid/ammonium oxalate extraction of the residue.*

*^g^Microwave-aided extraction.*

It should be mentioned that the high proportions of Glc found in some unpurified extracts are probably becoming from laminaran. This has occurred, for instance, in the sample of *Fucus serratus* isolated by Bittkau et al. (2020), as lower proportions of this monosaccharide have been found in other studies (Table 1). The studies of Bilan et al. (2002, 2004, 2006) on different *Fucus* species, carried out with careful separations involving anion exchange chromatography have shown in all cases that at high ionic strengths, they were able to isolate, with good yields, a fucan sulfate almost devoid of other monosaccharides (Fuc ≥ 94%, Table 1, fraction F_4_).

*Ascophyllum nodosum* is the other characteristic species from the family Fucaceae which has been thoroughly studied since the early studies of Kylin (1913), followed by further reports indicating the presence of a sulfated polysaccharide with a Fuc/Gal ratio of 8:1 (Percival and McDowell, 1967). The name ascophyllan was coined (to distinguish from the fucoidan characteristic of *Fucus vesiculosus*) for the isolated polysaccharide, composed of Fuc, Xyl, and sulfate groups, along with uronic acids. Medcalf and Larsen (1977a, b) determined a complex mixture of polysaccharides in this seaweed, and concluded that the fucan constituted the backbone of the molecule, whereas the ascophyllan-like components were attached as branches. Besides, they also determined that the uronic acid present was not glucuronic acid, as indicated in previous reports, but mannuronic and guluronic acid, i.e., the components of alginic acid, suggesting that contamination with this polysaccharide was difficult to avoid. For the fucoidans of this seaweed, an attempt was made to compare the results of the various researchers (Table 1), taking into account that most extractions were carried out in acid medium. However, the original Fuc/Xyl ratio close to 1 found by Larsen et al. (1966) was only reproduced by Nakayasu et al. (2009). Medcalf and Larsen (1977a) found a series of highly heterogeneous fractions, whereas 1 year later, using the same seaweed sample, Medcalf et al. (1978) found a polysaccharide with a Fuc/Gal ratio close to 1. The proportion of uronic acids in purified samples varied between 2 and 21%, whereas the content of sulfate varied between 8 and 24%. In summary, no common pattern between the determinations carried out by different researchers was observed.

Within the Fucaceae, it is clear that polysaccharides from the genus *Fucus* tend to be fucose-rich (more than 70% of the monosaccharides), although reports diverge, and important proportions of other monosaccharides appear in some cases (Table 1). On the other hand, in the genus *Ascophyllum*, important proportions of Xyl and uronic acid-containing fractions appear, although some purification steps allowed to obtained fucans equivalent to those of *Fucus*, suggesting that mixtures of different kinds of polymers appear in all the samples that have been surveyed in this study, and they might change their proportions in the different species, and using different extraction and purification methods.

The family Sargassaceae comprises much more species than the Fucaceae (512 against 18, Guiry and Guiry, 2020). This family has the largest number of species studied from the point of view of its polysaccharides. The fucoidans from at least 26 different species of the genus *Sargassum* alone were analyzed. Table 2 shows the results for the different fucoidans isolated from this genus. For *S. horneri*, Ermakova et al. (2011) postulated the presence of Rha in substantial amounts within the polysaccharides (Table 2). However, their NMR spectra did not show the presence of this sugar, and in a further work by the same group (Silchenko et al., 2017) the fucoidans were purified without any trace of Rha. In *S. latifolium*, Asker et al. (2007) isolated three fractions where Glc and GlcA are the major components and Fuc is a minor one, not responding to the classical fucoidan composition. Other atypical polysaccharides were reported in *S. pallidum* (Liu et al., 2016) carrying high-mannose fucoidans, rich in uronic acids and scarcely sulfated, and in *S. thunbergii* (Luo et al., 2019), where a fucoidan completely devoid of sulfate groups was reported (Table 2).

**TABLE 2:** Reported compositions of the fucoidans from the genus *Sargassum* (Sargassaceae, Fucales).

Species	Extraction	Purification/	Acronym	Monosaccharide composition (moles %)									Sulfate		UA (%)	References
		Fractionation^a^		Method^b^	Fuc	Xyl	Gal	Man	Glc	Rha	GlcA	Others	Method^c^	%		
*Sargassum aquifolium*	H_2_O + HCl pH 1	AEC	0.5M	GC	14	15	37	13	21				DP	6	28	Bilan et al. (2017)
	H_2_O + HCl pH 1	AEC	1M	GC	41	15	29	9	6				DP	22	14	“
	H_2_O + HCl pH 1	AEC	1.5M	GC	36	9	48	4	3				DP	29	5	“
*Sargassum binderi*	CaCl_2_ 2% hot	PQA	Fsar	GC	60	5	19	7			7	Ac	EA	8	^d^	Lim et al. (2016)
*Sargassum cinereum*	H_2_O+CaCl_2_ 1%			HPLC	66	7	24	3					DP	4	ND	Somasundaram et al. (2016)
*Sargassum crassifolium*	CaCl_2_ 2% hot	PQA	Fsc	GC	56	2	41	1					DP	28	8	Yuguchi et al. (2016)
	H_2_O, PT^e^	AP/R	SC3	PAD	37	5	37	11		11			IC	22	24	Yang et al. (2017)
*Sargassum duplicatum*	HCl 0.1M hot	AEC+HC	SdF1	GC	40		57	3				Ac	DP	32	ND	Shevchenko et al. (2017)
	HCl 0.1M hot	AEC+HC	SdF2	GC	59	2	39					Ac	DP	38	ND	“
	HCl 0.1M hot	AEC, NH_3_	SdF	GC	51		49					Ac	DP	32	ND	Usoltseva et al. (2017a)
*Sargassum feldmanii*	HCl 0.1M hot	AEC+HC	SfF2	GC	72		28						DP	25	ND	Shevchenko et al. (2017)
*Sargassum filipendula*	Enz.pH 8	Acetone ppt	SF-0.7	HPLC	22	16	27		16		16		DP	11	^d^	Costa et al. (2011)
	Enz.pH 8	Acetone ppt	SF-2.0	HPLC	22	4	49	13	11				DP	18		“
*Sargassum fulvellum*	HCl pH 2 hot	PQA	Fr 0.5	GC	38	23	26	6	7				DP	13	23	Koo et al. (2001)
	HCl pH 2 hot	PQA	Fr 3	GC	44	6	43	3	4				DP	55	4	“
*Sargassum fusiforme*	H_2_O, hot	AEC+SEC	SFPS	GC	53	9	20	21					DP	11	6	Chen et al. (2012)
	Enzymes	AP/R+SEC	65A	GC	42	15	21	6	2		14		DP	17	^d^	Hu et al. (2016)
	H_2_O+CaCl_2_ 2%	AEC+SEC	FP08S2	GC	37	18	19	7			19		EA	21	^d^	Cong et al. (2016)
	HCl 0.01M+CaCl_2_ 4M	AEC+SEC	SFF42	HPLC	31	6	19	29	3	12			DP	17	12	Wu et al. (2019)
	HCl 0.01M+CaCl_2_ 4M	AEC+SEC	SFF5	HPLC	50	3	31	10	3	3			DP	24	10	“
*Sargassum hemiphyllum*	H_2_O, PT^e^	CaCl_2_ 2%+AP/R	SH3	PAD	54	1	19	15	3	8		Ac	IC	24	6	Huang et al. (2017)
*Sargassum henslowianum*	H_2_O, AP/R	AEC+SEC	SHAP-1	HPLC	76		24						EA	32	0	Sun et al. (2020)
	H_2_O, AP/R	AEC+SEC	SHAP-2	HPLC	75		25						EA	32	0	“
*Sargassum horneri*	HCl 0.1M hot	AEC	Sh-F1	HPLC	81	3	8			7			DP	15	ND	Ermakova et al. (2011)
	HCl 0.1M hot	AEC	Sh-F2	HPLC	90					10			DP	0	ND	“
	HCl 0.1M hot	AEC	Sh-F3	HPLC	69					31			DP	17	ND	“
	CaCl_2_ 2% hot	AEC		GC	90		10						DP	23	ND	Silchenko et al. (2017)
*Sargassum latifolium*	H_2_O, hot	AEC+SEC	SP-I	HPLC	14	14			42		23			16	^d^	Asker et al. (2007)
	H_2_O, hot	AEC+SEC	SP-II	HPLC	10	13			41		29			19	^d^	“
	H_2_O, hot	AEC+SEC	SP-III	HPLC	16	12			32		35			22	^d^	“
*Sargassum mcclurei*	HCl pH 2.5 hot	HC+AEC	SmF1	HPLC	27	6	20	34	13				DP	17	ND	Thinh et al. (2013)
	HCl pH 2.5 hot	HC+AEC	SmF2	HPLC	45	5	34	5	10				DP	26	ND	“
	HCl pH 2.5 hot	HC+AEC	SmF3	HPLC	59		41						DP	35	ND	“
*Sargassum muticum*	pH 7.5+CaCl_2_ 1%	EtOH+TCA 10%	FF	GC	44	5	46	3	3				Tit	12	9	Mabeau et al. (1990)
	Triton 0.5%, pH 7.5+CaCl_2_ 1%	EtOH+TCA 10%	TF	GC	84	2	14						Tit	8	11	“
	HCl 0.01M+CaCl_2_ 1%		HF	GC	46	21	11	17	5				Tit	9	25	“
	HCl 0.1M hot	AEC	1SmF1	GC	52		33	15					DP	26	ND	Usoltseva et al. (2017b)
	HCl 0.1M hot	AEC	1SmF3	GC	67		33					Ac	DP	48	ND	“
*Sargassum oligocystum*	HCl 0.1M hot	AEC	1SoF1	HPLC	43	4	8	35	8				DP	17	ND	Men’shova et al. (2013)
	HCl 0.1M hot	AEC	1SoF2	HPLC	53	5	21	10	10				DP	24	ND	“
	HCl 0.1M hot	AEC	1SoF3	HPLC	77		23						DP	32	ND	“
*Sargassum pallidum*	HCl 0.2M hot		Sterile	HPLC	46	8	10	10	14	13				ND	ND	Skriptsova et al. (2012)
	HCl 0.2M hot		Reprod.	HPLC	52	6	16	9	3	14				ND	ND	“
	H_2_O, r.t.	Ethanol ppt	SPC60	GC	41	5	17	27	10				DP	4	33	Liu et al. (2016)
	H_2_O, hot	Ethanol ppt	SPH60	GC	32	4	14	23	25				DP	4	29	“
	H_2_O, hot	Ethanol ppt	SPH70	GC	37	4	24	22	10				DP	7	20	“
*Sargassum polycystum*	HCl pH 2-3 hot	HC+AEC	F1	GC	29	22	19	19	11				DP	7	23	Bilan et al. (2013)
	HCl pH 2-3 hot	HC+AEC	F2	GC	44	13	28	9	5				DP	20	11	“
	HCl pH 2-3 hot	HC+AEC	F3	GC	69	4	25	tr.	tr.				DP	33	2	“
	HCl pH 2-3 hot	HC+AEC	F4	GC	63	3	34						DP	34	2	“
	Enzymes pH 4.5	CaCl_2_ 5M	SPF	PAD	63	6	8					NI^f^ 22	DP	28		Fernando et al. (2018)
*Sargassum ringgoldianum*	HCl 0.05M	Ca(AcO)_2_+AEC	Fr-B	GC	44	17	18	17		5			DP	16	10	Mori and Nisizawa (1982)
	HCl 0.05M	Ca(AcO)_2_+AEC	Fr-C	GC	58	6	28	7		1			DP	24	7	“
*Sargassum stenophyllum*	H_2_O+CaCl_2_ 4M	PQA	F2	GC	60	9	21	10					DP	19	11	Duarte et al. (2001)
	H_2_O+CaCl_2_ 4M	PQA	F3	GC	52	7	23	17					DP	21	10	“
	H_2_O+CaCl_2_ 4M	PQA	F5	GC	60	5	31	2	2				DP	28	2	“
*Sargassum swartzii*	HCl 0.1M +CaCl_2_ 2%	PQA+AEC	F2	PAD	50	3	29	5	3			Ara 7	DP	15	13	Ly et al. (2005)
	HCl 0.1M +CaCl_2_ 2%	PQA+AEC	F3	PAD	56	2	29	3	3			Ara 5	DP	18	5	“
	HCl 0.1M +CaCl_2_ 2%	PQA+AEC	F4	PAD	56	2	28	4	3			Ara 4	DP	28	8	“
	HCl 0.05 M+CaCl_2_ 4%	AEC	FF1	HPLC	58	6	22	14					DP	19	18	Dinesh et al. (2016)
	HCl 0.05 M+CaCl_2_ 4%	AEC	FF2	HPLC	63	4	18	15					DP	24	13	“
*Sargassum tenerrimum*	HCl 0.1M +K_2_CO_3_ 2%	CaCl_2_ 2%+ HCl 0.1M	C	GC	73	15	9		3				DP/IR	2	9	Sinha et al. (2010)
*Sargassum trichophyllum*	H_2_O, hot	AEC+SEC	ST-F	GC	80		20						Rho	23	1	Lee et al. (2011)
*Sargassum thunbergii*	H_2_O+NaOH 0.5M	AEC	STSP-I	GC	55		45						DP	0	ND	Luo et al. (2019)
*Sargassum vachellianum*	H_2_O	CaCl_2_	SPS	HPLC	65	5	12	15	3				DP	12	1	Jesumani et al. (2020)
*Sargassum vulgare*	Enz. pH 8	AEC	Flo 1.5	Col.	50^g^	25						HexA 25	TB	∼ 15	^d^	Dietrich et al. (1995)
	Enz. pH 8	AEC	Flo 2.5	Col.	77^g^	8						HexA 15	TB	∼ 41	^d^	“

*^*a*^Key: AEC, anion exchange chromatography; SEC, size-exclusion chromatography; HC, hydrophobic chromatography; PQA, precipitation with quaternary ammonium salts; AP/R alcohol precipitation and redissolution.*

*^b^Key for the less common abbreviations: PAD, HPAEC with pulse amperometric detector; GC, gas chromatography; Col., colorimetric methods.*

*^c^Key: DP, method of Dodgson and Price (1962) or equivalent; IC, ion chromatography; EA, elemental analysis; IR, estimation by area of IR bands; TB, toluidine blue; Rho, rhodizonate; Tit, titration with cetylpyridinium chloride, pH 1.5 (Scott, 1960).*

*^d^The information for the uronic acid is included in the molar ratio of monosaccharides. ^*e*^PT = high pressure and temperature.*

*^f^NI = sugar not identified.*

*^g^Fuc, Xyl and uronic acid were the only monosaccharides which could be determined.*

Dietrich et al. (1995) studied the polysaccharides from *Sargassum vulgare*, differentiating whole plants and floaters. The fucoidan fractions corresponded to sulfated xylofucans containing important proportions of uronic acids. The proportion of sulfate is clearly higher in floaters. The ratio Fuc/Xyl/HexA varied between 1:0.5:0.5 and 1:0.1:0.2. However, only Fuc, Xyl and uronic acid have been determined in this investigation, missing other sugars possibly present.

For *Sargassum fusiforme*, the presence of galacturonic acid was detected (Hu et al., 2014). However, it has been shown later that this monosaccharide was part of a contaminating polysaccharide which could be separated by careful fractionation (Cong et al., 2016; Hu et al., 2016).

For the remaining members of the Fucales, the data is shown in Table 3. Mian and Percival (1973) carried out studies on *Bifurcaria bifurcata* and *Himanthalia lorea.* The data is shown only partially in Table 3, as Gal could not be quantified. Fractionation by ion exchange chromatography showed fractions with high uronic acid/low sulfate content using lower ionic strengths, and high sulfate, high Fuc, low uronic acid content in the later elutions. This behavior was observed for many further studies, regardless of the taxonomy of the seaweed. In some cases, like for *Nizamuddinia zanardinii*, the authors have devoted a lot of work in order to search for different extraction methods (Alboofetileh et al., 2019a,b,c). In Table 3 we have included the analysis of one extraction method, as the characteristics of the polysaccharides appear to be quite similar.

**TABLE 3:** Reported compositions of the fucoidans from the order Fucales not belonging to the family Fucaceae or to the genus *Sargassum* (Sargassaceae).

Species	Extraction	Purification/	Acronym	Monosaccharide composition (moles %)									Sulfate		UA (%)	References
		Fractionation^a^		Method^b^	Fuc	Xyl	Gal	Man	Glc	Rha	GlcA	Others	Method^c^	%		
**Family Sargassaceae**																
*Bifurcaria bifurcata*	CaCl_2_ 2% +HCl pH2	AEC	0.3M	GC+PC	XX	X	tr^e^						JL	5	20	Mian and Percival (1973)
	CaCl_2_ 2% +HCl pH2	AEC	1M	GC+PC	XX	tr.	X^e^						JL	30	3	“
	HCl 0.01M+CaCl_2_ 1%			GC	73	10	10	4	3				Tit	20	16	Mabeau and Kloareg (1987)
*Coccophora langsdorfii*	HCl 0.1M r.t.	AEC	Cf2	HPLC	86	3	7					HexA 4,Ac	DP	25	^d^	Imbs et al. (2016)
*Cystoseira barbata*	HCl 0.1M hot		CBSP	GC	45	4	34	3	8	6		Ac	EA	23	7	Sellimi et al. (2014)
*Cystoseira compressa*	HCl 0.1M hot		CCF	GC	62	4	24		8				DP	15	9	Hentati et al. (2018)
*Cystoseira indica*	H_2_O, r.t.		CiWE	GC	75	14	11						DP/IR	8	4	Mandal et al. (2007)
	H_2_O, r.t.	AEC	CiF3	GC	84	7	5	4					DP/IR	9	2	“
*Hizikia fusiforme*	H_2_O+CaCl_2_ 3M	AEC	F2	GC	38	8	18	30	4	1			DP	12	29	Li et al. (2006)
	H_2_O+CaCl_2_ 3M	AEC+SEC	F33	GC	38	5	22	27	5	2			DP	3	32	“
	H_2_O+CaCl_2_ 3M	AEC	YF5	HPLC	44		21	18			16		DP	20	^d^	Wang et al. (2012)
*Hormophysa cuneiformis*	H_2_O+HCl pH 1		FHC	GC	39	5	47	5	4				DP	23	5	Bilan et al. (2018)
	H_2_O+HCl pH 1	AEC	F2	GC	33	11	50	4	2				DP	18	7	“
	H_2_O+HCl pH 1	AEC	F3	GC	79	2	19						DP	35	2	“
*Nizamuddinia zanardinii*	H_2_O	CaCl_2_ 1%	HWE-F	GC	31	6	28	32	5				DP	18	1	Alboofetileh et al. (2019a)
*Turbinaria conoides*	HCl 0.1M	AEC	AF3	GC	54	18	28				+		DP/IR	4	ND	Chattopadhyay et al. (2010)
*Turbinaria ornata*	HCl 0.1M hot	AEC	ToF2	HPLC	83		17						DP	32	ND	Ermakova et al. (2016)
	Enzymes pH 4.5	CaCl_2_+AEC	F2	PAD	46		22					NI^f^ 32	DP	10	ND	Jayawardena et al. (2019)
	Enzymes pH 4.5	CaCl_2_+AEC	F7	PAD	63		5	6				NI 25	DP	30	ND	“
*Turbinaria turbinata*	Enzymes pH 5	AEC	TtF3	GC	61	2	19	4	13			Ara 1,Ac		ND	ND	Monsur et al. (2017)
**Family Durvillaeaceae**																
*Durvillaea antarctica*	H_2_O, MW^g^		DAP	GC	3	3		9	78			Sorbose 8		ND	ND	He et al. (2016)
*Durvillaea potatorum*	HCl pH 1 hot	Acetone ppt	AFS	HPLC	32		4		64				DP	13	–	Lorbeer et al. (2017)
**Family Himanthaliaceae**																
*Himanthalia elongata*	H_2_O+HCl 0.1M		F-HCl	GC	17	1	29	3	50				DP	6	3	Mateos-Aparicio et al. (2018)
*Himanthalia lorea*	CaCl_2_ 2% +HCl pH2	AEC	0.3M	GC+PC	XX	X	tr.^e^						JL	2	19	Mian and Percival (1973)
	CaCl_2_ 2% +HCl pH2	AEC	1M	GC+PC	XX	tr.	X^e^						JL	29	4	“
**Family Seirococcaceae**																
*Marginariella boryana*	H_2_SO_4_ 1% r.t.	Reprod.	GC	72	2	17	1	7					ND	3	Wozniak et al. (2015)
	H_2_SO_4_ 1% r.t.	Vegetat.	GC	45	21	12	13	7	2				ND	13	“
*Seirococcus axillaris*	HCl pH 1 hot	Acetone ppt	AFS	HPLC	61	16	14	3	2		4		DP	20	^d^	Lorbeer et al. (2017)

*^a^Key: AEC, anion exchange chromatography; SEC, size-exclusion chromatography.*

*^b^Key for the less common abbreviations: PC, paper chromatography; GC, gas chromatography; PAD, HPAEC with pulse amperometric detector.*

*^c^Key DP, method of Dodgson and Price (1962) or equivalent; JL, method of Jones and Letham (1954); IR, estimation by area of IR bands; EA, elemental analysis by different methods; Tit, titration with cetylpyridinium chloride, pH 1.5 (Scott, 1960).*

*^d^The information for the uronic acid is included in the molar ratio of monosaccharides.*

*^e^As galactose could not be quantified, the data is semiquantitative.*

*^f^NI = sugar not identified.*

*^g^Microwave-aided extraction.*

For *Marginariella boryana*, Wozniak et al. (2015) analyzed the polysaccharides extracted from vegetative structures (blades and vesicles) and receptacles (reproductive structures) separately. The proportions of Xyl, Man, and uronic acid increase significantly in the vegetative structures (Table 3). Within the family Durvillaeaceae two species were studies. Both in *Durvillaea antarctica* (He et al., 2016) and *D. potatorum* (Lorbeer et al., 2017), the proportion of Glc was so large that it obscured the analysis of the fucoidan constituents, even when purification procedures (successful with other seaweeds) to avoid contamination with laminaran were carried out (Lorbeer et al., 2017).

Most of the fucoidans analyzed from the Fucales were galactofucans, usually with small proportions of Xyl, with the exception of those of *Ascophyllum nodosum* (Table 1). Man and GlcA appeared in variable amounts.

### Dictyotales

The data on the fucoidans from different species of the order Dictyotales is shown in Table 4. It should be mentioned that for *Dictyota mertensii*, the information is incomplete, as only Fuc, Xyl and uronic acid have been determined (Dietrich et al., 1995).

**TABLE 4:** Reported compositions of the fucoidans from the order Dictyotales.

Species	Extraction	Purification/	Acronym	Monosaccharide composition (moles %)									Sulfate		UA (%)	References
		Fractionation^a^		Method^b^	Fuc	Xyl	Gal	Man	Glc	Rha	GlcA	Others	Method^c^	%		
*Canistrocarpus cervicornis*	Enz.pH 8	Acetone ppt	CC-0.7	HPLC	33	17					50		DP	19	^d^	Camara et al. (2011)
	Enz.pH 8	Acetone ppt	CC-2.0	HPLC	20	10	40	10			20		DP	20	^d^	“
*Dictyopteris plagiogramma*	CaCl_2_ 2% +HCl pH2		C	GC	42	10	16	8	3		21		JL	4	^d^	Percival et al. (1981)
*Dictyopteris polypodioides*	HCl 0.1M hot	HC+AEC	Dp-F2	HPLC	48	19	5	14	5	9			DP	13	ND	Sokolova et al. (2011)
	HCl 0.1M hot	HC+AEC	Dp-F4	HPLC	38	8	31	4	8	12			DP	13	ND	“
*Dictyota dichotoma*	HCl pH 1 hot	Ethanol ppt	R	PC	25	16	25	10			24		BC	16	^d^	Abdel-Fattah et al. (1978)
	HCl pH 2 r.t.	PQA	EAR-0.5	GC	40	30	6	16	4				DP	13	40	Rabanal et al. (2014)
	HCl pH 2 r.t.	PQA	EAR-2	GC	43	16	28	10	2				DP	33	14	“
	HCl pH 2 hot	PQA	EAH1-1.5	GC	41	26	5	25	1	2			DP	19	30	“
	HCl pH 2 hot	PQA	EAH2-0.5	GC	26	36	4	33		1			DP	10	42	“
	HCl pH 2 hot	PQA	EAH4-0.5	GC	10	30	5	51	3				DP	5	48	“
	HCl 0.1M hot	AEC+HC	DdF	GC	52	12	10	9	17			Ac	DP	2	ND	Shevchenko et al. (2017)
	HCl 0.1M hot	AEC (x 2)	DdF	HPLC	58		20	12	9			Ac	DP	29	ND	Usoltseva et al. (2018b)
*Dictyota divaricata*	HCl 0.1M hot	AEC+HC	DdiF1	GC	61		31	4	4			Ac	DP	11	ND	Shevchenko et al. (2017)
	HCl 0.1M hot	AEC+HC	DdiF2	GC	43	5	44	4	4				DP	18	ND	“
*Dictyota menstrualis*	Enz. pH 8	Acetone ppt	F1.0v	PC+GC	30	24	24					HexA 21		∼ 5	^d^	Albuquerque et al. (2004)
	Enz. pH 8	Acetone ppt	F1.5v	PC+GC	31	9	47					HexA 13		∼ 16	^d^	“
*Dictyota mertensii*	Enz. pH 8	AEC	1M	Col.	26^e^	32						HexA 42	TB	∼ 20	^d^	Dietrich et al. (1995)
	Enz. pH 8	AEC	2.5+3M	Col.	56^e^	11						HexA 33	TB	∼ 37	^d^	“
	Enz. pH 8	Acetone ppt	ADm	GC	33	20					47		DP	∼ 22	^d^	Queiroz et al. (2008)
*Lobophora variegata*	Enz. pH 8	Acet + SEC	Lv	GC	25		75					Ac	DP	∼ 3	–	Medeiros et al. (2008)
*Padina australis*	CaCl_2_ 2% hot	PQA	Fpa	GC	60	8	29	3					DP	22	21	Yuguchi et al. (2016)
*Padina boryana*	HCl 0.1M hot	AEC+HC	PbF	GC	61		31	4	3			Ac	DP	18	ND	Shevchenko et al. (2017)
	HCl 0.1M hot	AEC (x 2)	PbF	GC	40		37	17	6			Ac	DP	19	ND	Usoltseva et al. (2018a)
*Padina gymnospora*	Enz. pH 8	Acet + SEC	PF1	PC+GC	36	11	7				46		DP	6	^d^	Silva et al. (2005)
	Enz. pH 8	Acet + SEC	PF2	PC+GC	39	8	6				47		DP	3	^d^	“
*Padina pavonica*	CaCl_2_ 2% +HCl pH2	AEC	0.3M	PC+GC	XX	X	tr.^f^						JL	3	20	Mian and Percival (1973)
	CaCl_2_ 2% +HCl pH2	AEC	1M	PC+GC	XX	tr.	X^f^						JL	17	5	“
	HCl pH 2.5 hot	AEC	Purified	PC	16	16	11	13	13		30		BC	19	^d^	Hussein et al. (1980)
	HCl 0.1M hot	AEC	4PpF1	HPLC	43	13	9	17	17				DP	4	ND	Men’shova et al. (2012)
	HCl 0.1M hot	AEC	4PpF2	HPLC	53	16	16	10		5			DP	14	ND	“
	HCl 0.1M hot	AEC	4PpF3	HPLC	59	6	18			18			DP	18	ND	“
*Padina tetrastomatica*	H_2_O	CaCl_2_ 2% ppt	PtWE1	GC	59	23	10	3		5				ND	9	Karmakar et al. (2009)
	H_2_O	AEC+SEC	F3	GC	72	25	3						DP/IR	∼ 8	4	“
	HCl 0.1M r.t.		Ext. A	GC	68	16	9	5	2				DP/IR	∼ 3	5	Karmakar et al. (2010)
	HCl 0.1M +K_2_CO_3_ 2%	CaCl_2_ 2% ppt	Ext. C	GC	73	16	11						DP/IR	∼ 6	5	“
*Spatoglossum asperum*	H_2_O+CaCl_2_ 1%	AP/R		HPLC	61	6	25	4		3			DP	21	ND	Palanisamy et al. (2017)
*Spatoglossum schroederi*	Enz. pH 8	Acetone ppt	Fuc. A	GC	53	18					29		DP	∼ 28	^d^	Queiroz et al. (2008)
	Enz. pH 8	Acetone ppt	Fuc. B	GC	27	14	55				4		DP	∼ 37	^d^	“
	Enz. pH 8	Acet.+AEC	Fuc. B	GC	28	14	56				2		TB	19	^d^	Menezes et al. (2018)
*Stoechospermum marginatum*	H_2_O	AEC (x 2)	F3	GC	96	2	2						DP/IR	13	–	Adhikari et al. (2006)

*^a^Key: AEC, anion exchange chromatography; SEC, size-exclusion chromatography; HC, hydrophobic chromatography; PQA, precipitation with quaternary ammonium salts; Acet, fractional precipitation with acetone; AP/R alcohol precipitation and redissolution.*

*^b^Key for the less common abbreviations: PC, paper chromatography; GC, gas chromatography; Col., colorimetric methods.*

*^c^Key DP, method of Dodgson and Price (1962) or equivalent; JL, method of Jones and Letham (1954); BC, method of barium chloranilate (Lloyd, 1959); TB, method of toluidine blue; IR, estimation by area of IR bands.*

*^d^The information for the uronic acid is included in the molar ratio of monosaccharides.*

*^e^Fuc, Xyl and uronic acid were the only monosaccharides which could be determined.*

*^f^As galactose could not be quantified, the data is semiquantitative.*

*Padina pavonica* was studied by Mian and Percival (1973), named then as *P. pavonia*. As occurred with the other seaweeds studied in that paper, the data on the table are incomplete, as Gal could not be quantified. Fraction 0.3M was rich in Fuc and Xyl, whereas fraction 1M was richer in Fuc, together with Gal. For this seaweed, Men’shova et al. (2012) carried out a seasonal study which showed that the proportion of Gal of the fucoidans increased markedly in all fractions when stepping down from spring to summer.

The fucoidans from the Dictyotales appear to be more heterogeneous than most of those of the Fucales. High proportions of Man and Rha appeared often (Table 4). However, an almost pure fucan sulfate was reported to be present in *Stoechospermum marginatum* (Adhikari et al., 2006) after careful purification.

### Laminariales

Two species of Laminariales have been included in the early studies of Kylin (1913). They are *Laminaria digitata* and *Saccharina lattisima* (as *Laminaria saccharina*).

Many different species from the Laminariales have been studied thereafter, including species from four families (Agaraceae, Alariaceae, Laminariaceae, and Lessoniaceae). In order to keep up with the Silberfeld et al. (2014) taxonomy, we have included also a species from the *Chorda* genus (family Chordaceae) which has been recently proposed to be included in a new order, the Chordales (Starko et al., 2019). The data for the family Laminariaceae are shown in Table 5, whereas those of the remaining families appear in Table 6. It is worth noting that the species studied as *Laminaria cichorioides* and *L. japonica* are included in Table 5 as *Saccharina cichorioides* and *S. japonica*, respectively, in order to keep up with the newer taxonomy (Guiry and Guiry, 2020).

**TABLE 5:** Reported compositions of the fucoidans from the family Laminariaceae (order Laminariales).

Species	Extraction	Purification/	Acronym	Monosaccharide composition (moles %)									Sulfate		UA (%)	References
		Fractionation^a^		Method^b^	Fuc	Xyl	Gal	Man	Glc	Rha	GlcA	Others	Meth.^c^	%		
*Kjelmaniella crassifolia*	pH 6.5 hot	HCl pH 2 ppt		HPLC	84		5	10						ND	7	Sakai et al. (2002)
	Enz. pH 4.5	AEC	F1	HPLC	30	3	49	6	4		9	Ac	DP	23	^d^	Song et al. (2018)
	Enz. pH 4.5	AEC	F2	HPLC	47	8	15	12	1		16	Ac	DP	16	^d^	“
	Enz. pH 4.5	AEC	F3	HPLC	67	2	23	3	1		4		DP	32	^d^	“
*Laminaria angustata*	H_2_O	PQA+AEC	F4	GC	90		10						EA	∼22	1	Kitamura et al. (1991)
	HCl pH 2 +PQA	AEC+SEC	LA-5	GC	2		98						DP	38	3	Nishino et al. (1994b)
	HCl 0.1M	PQA+AEC	LA-2	PAD	95	5							DP	56	2	Tako et al. (2010)
*Laminaria bongardiana*	CaCl_2_ 2% hot	PQA+AEC	F-2	GC	53	8	20	15	3			Ac	DP	20	12	Bilan et al. (2016)
	CaCl_2_ 2% hot	PQA+AEC	F-3	GC	39	4	54	2	1			Ac	DP	26	3	“
*Laminaria cichorioides*	**See *Saccharina cichorioides***															
*Laminaria digitata*	HCl 0.01M+CaCl_2_ 1%			GC	62	21	9	4	4				Tit	9	15	Mabeau and Kloareg (1987)
	pH 7.5+CaCl_2_ 1%	EtOH+TCA 10%	FF	GC	65	4	24	3	4				Tit	18	7	Mabeau et al. (1990)
	Triton 0.5%, pH 7.5+CaCl_2_ 1%	EtOH+TCA 10%	TF	GC	47	15	20	11	7				Tit	11	12	“
	CaCl_2_ 2% hot	PQA		GC	73	5	15	4	3				DP	27	7	Cumashi et al. (2007)
	CaCl_2_ 2% hot			GC	67	14	14	5					EA	20	10	Bittkau et al. (2020)
*Laminaria hyperborea*	Exudation	UF	pFuc	GC	98		2		tr.				EA	54	–	Kopplin et al. (2018)
*Laminaria japonica*	**See *Saccharina japonica***															
*Laminaria longipes*	HCl 0.1M r.t.	AEC	LlF	GC	100								DP	32	ND	Usoltseva et al. (2019)
*Laminaria religiosa*	HCl pH 2 hot	PQA	Fr 0.5	GC	34	12	14	21	19				DP	9	35	Koo et al. (2001)
	HCl pH 2 hot	PQA	Fr. 3	GC	61	1	28	7	3				DP	39	18	“
*Macrocystis pyrifera*	Exudation	AP/R		PC+CC	92	2	6	tr.						19	–	Schweiger (1962)
			SigmaTM	HPLC	79	3	12	3	3				DP	27	5	Zhang et al. (2015)
	HCl pH 1 hot	Acetone ppt	AFS	HPLC	80		17	3					DP	24	–	Lorbeer et al. (2017)
*Saccharina cichorioides*	HCl 0.4%+H_2_O	HC	L.c.F-2	HPLC	81	2	4	2	3	8			DP	∼35	ND	Zvyagintseva et al. (1999)
	HCl 0.4% r.t.	HC	Lc2-F1	HPLC	72	7	8	8	5				DP	∼30	ND	Zvyagintseva et al. (2003)
	HCl 0.4% +H_2_O	HC	Lc2-F2	HPLC	100								DP	∼36	ND	“
	HCl pH 2-2.3 hot	AEC	Lc-F2	HPLC	98			2					DP	30	ND	Anastyuk et al. (2010)
	HCl 0.1M r.t.	AEC	Sc-F1	HPLC	95			5					DP	21	ND	Vishchuk et al. (2013)
	HCl 0.1M r.t.	AEC	Sc-F2	HPLC	100								DP	39	ND	“
	HCl pH 2-2.3	AEC	ScF	HPLC	89	2	6	3					DP	26	ND	Prokofjeva et al. (2013)
	HCl 0.1M r.t.	AEC	ScF	GC	98		2						DP	36	ND	Usoltseva et al. (2019)
*Saccharina gurjanovae*	HCl pH 2-2.3	AEC	SgGF	HPLC	64		21	15				Ac	DP	28	ND	Prokofjeva et al. (2013)
	CaCl_2_ 2% hot	AEC (x 2)	SgF	GC	76		24					Ac	DP	25	ND	Shevchenko et al. (2015)
*Saccharina japonica*	HCl 0.4% +H_2_O	HC	L.j.-F-2	HPLC	94	2	3	1						ND	ND	Zvyagintseva et al. (1999)
	HCl 0.4% r.t.	HC	Lj1-F1	HPLC	55	7	26	6	3	3				ND	ND	Zvyagintseva et al. (2003)
	HCl 0.4% +H_2_O	HC	Lj1-F2	HPLC	84	1	12		1	2			DP	∼25	ND	“
	HCl pH 3 r.t.	AEC	L	HPLC	61	5	14	16	4				DP	21	18	Ozawa et al. (2006)
	HCl pH 3 r.t.	AEC	GA	HPLC	90		10						DP	38	1	“
	HCl 0.1M hot	AEC	Sj-F1	HPLC	53	1	29	15		2			DP	10	ND	Vishchuk et al. (2011)
	HCl 0.1M hot	AEC	Sj-F2	HPLC	61	2	33	1		3		Ac	DP	23	ND	“
	HCl 0.2M hot		Sterile	HPLC	41	8	14	12	14	11				ND	ND	Skriptsova et al. (2012)
	HCl 0.2M hot		Reprod.	HPLC	25	3	13	4	48	7				ND	ND	“
	HCl 0.1M hot	AEC	Sj-sF2	HPLC	62	6	21	9		2			DP	21	ND	Vishchuk et al. (2012)
	HCl 0.1M hot	AEC	Sj-fF2	HPLC	58		37	5					DP	23	ND	“
	HCl pH 2-2.3	AEC	SjGF	HPLC	50	1	44	5				Ac	DP	23	ND	Prokofjeva et al. (2013)
	HCl pH 2.5 hot		B	CZE	54	3	29	3		1	10			ND	^d^	Guo et al. (2013)
	H_2_O hot	CaCl_2_ 1%+AP/R	LJF	HPLC	34	2	37	23	1	3			DP	14	3	Qu et al. (2014)
	HCO_2_H 0.1%, PT^e^	CaCl_2_ 1%		HPLC	57		17	21	5				DP	24	10	Saravana et al. (2016)
*Saccharina latissima*	CaCl_2_ 2% hot	PQA		GC	80	3	10	2	5				DP	30	5	Cumashi et al. (2007)
	CaCl_2_ 2% hot	PQA+AEC	F-1.0	GC	46	5	32	14	3				DP	16	23	Bilan et al. (2010)
	CaCl_2_ 2% hot	PQA+AEC	F-1.25	GC	78	2	18	2					DP	37	2	“
	CaCl_2_ 2% hot	AEC	B06-F2	GC	56	14	14	13	3				EA	6	–	Ehrig and Alban (2015)
	CaCl_2_ 2% hot	AEC	B06-F3	GC	76	3	20	1					EA	16	–	“
	CaCl_2_ 2% hot			GC	84	7	7			2			EA	29	6	Bittkau et al. (2020)
	Enz.pH6 + CaCl_2_ 2%	AEC	SlF3	PAD	63	3	27	2				HexA 4	DP	46	^d^	Nguyen et al. (2020)
*Saccharina longicruris*	CaCl_2_ 2% +HCl 0.01M		B		ND								EA	14	8	Rioux et al. (2007)

*^a^Key: AEC, anion exchange chromatography; SEC, size-exclusion chromatography; HC, hydrophobic chromatography; PQA, precipitation with quaternary ammonium salts; AP/R alcohol precipitation and redissolution; UF, ultrafiltration.*

*^b^Key for the less common abbreviations: PAD, HPAEC with pulse amperometric detector; PC, paper chromatography; GC, gas chromatography; CC, column chromatography on cellulose; CZE, capillary zone electrophoresis.*

*^c^Key DP, method of Dodgson and Price (1962) or equivalent; EA, elemental analysis by different methods; Tit, titration with cetylpyridinium chloride, pH 1.5 (Scott, 1960).*

*^d^The information for the uronic acid is included in the molar ratio of monosaccharides.*

*^e^High pressure and temperature have been applied.*

**TABLE 6:** Reported compositions of the fucoidans from the order Laminariales (families other than the Laminariaceae).

Species	Extraction	Purification/	Acronym	Monosaccharide composition (moles %)									Sulfate		UA (%)	References
		Fractionation^a^		Method^b^	Fuc	Xyl	Gal	Man	Glc	Rha	GlcA	Others	Method^c^	%		
**Family Agaraceae**																
*Costaria costata*	HCl pH 2-2.3 hot		FLM7	HPLC	62	4	18	5	7	4			DP	12	ND	Imbs et al. (2009)
	HCl 0.1M hot	AEC	CcF	HPLC	51	3	43	tr.		3		Ac	DP	19	ND	Ermakova et al. (2011)
	HCl pH 2-2.3 r.t.	HC	F1.5	HPLC	70		20	7			3		DP	24	^d^	Imbs et al. (2011)
	HCl pH 2-2.3 hot	AEC	5F2	GC	30	16	8	15			15		DP	15	^d^	Anastyuk et al. (2012a)
	HCl pH 2-2.3 hot	AEC	5F3	GC	40	12	21	12	6		7		DP	15	^d^	“
	HCl pH 2-2.3		CcGF	HPLC	63		30	3		2		Ac	DP	23	ND	Prokofjeva et al. (2013)
	Enz. pH 4.5	AP/R+AEC	F2	GC	17	7	8	61	8				Grav	1	ND	Wang et al. (2014)
	Enz. pH 4.5	AP/R+AEC	F4	GC	47	17	17	12	8				Grav	23	ND	“
	Enz. pH 4.5	AEC	6F1	GC	21	11	20	30	7	10			DP	9	4	Liu et al. (2018)
	Enz. pH 4.5	AEC	6F2	GC	31	15	9	26	11	8			DP	10	6	“
**Family Alariaceae**																
*Alaria angusta*	HCl 0.1M hot	HC+AEC	AaF2	HPLC	75		7	18					DP	14	ND	Menshova et al. (2015)
	HCl 0.1M hot	HC+AEC	AaF3	HPLC	53		47					Ac	DP	24	ND	“
*Alaria marginata*	HCl 0.1M hot	HC+AEC	AmF2	HPLC	81		9	11					DP	21	ND	Usoltseva et al. (2016)
	HCl 0.1M hot	HC+AEC	AmF3	HPLC	48	5	47					Ac	DP	28	ND	“
*Alaria ochotensis*	HCl 0.2M hot		Sterile	HPLC	18	4	10	4	59	6				ND	ND	Skriptsova et al. (2012)
	HCl 0.2M hot		Reprod.	HPLC	25	3	23	5	40	4				ND	ND	“
	HCl pH 2-2.3	AEC	AoGF	HPLC	54		38	8					DP	24	ND	Prokofjeva et al. (2013)
*Undaria pinnatifida*	HCl 0.15M	AEC+SEC	CF-4B	GC	48		52						EA	32	2	Lee et al. (2004)
	H_2_SO_4_ 1% r.t.	AEC	F2M	GC	54		45			1			EA	∼ 28	1	Hemmingson et al. (2006)
	HCl 0.2M hot	UF	F > 30K	HPLC	64		32	4					DP	32	ND	You et al. (2010)
	HCl 0.1M r.t.	AP/R+AEC		GC	51	4	45					Ac	EA	30	ND	Synytsya et al. (2010)
	HCl 0.1M hot	AEC	Up-F1	HPLC	59	2	30	8	1				DP	14	ND	Vishchuk et al. (2011)
	HCl 0.1M hot	AEC	Up-F2	HPLC	51		48	1				Ac	DP	29	ND	“
	CaCl_2_ 2% hot	PQA+AEC	F1	GC	49	4	38	7	3				DP	7	4	Mak et al. (2013)
	CaCl_2_ 2% hot	PQA+AEC	F3	GC	60	2	29	7	3				DP	25	1	“
	HCl 0.2M r.t.			GC	53		42	2	3					ND	2	Wozniak et al. (2015)
			SigmaTM	PAD	55		45						DP	26	2	Lu et al. (2018)
	H_2_O+CaCl_2_ 2%	SEC	F300	HPLC	56	7	35		2				DP	20	5	Koh et al. (2019)
**Family Chordaceae^e^**																
*Chorda filum*	CaCl_2_ 2% hot	AEC	A-2	GC	95	1	1	1	2			Ac	DP	26	–	Chizhov et al. (1999)
	Na_2_CO_3_ 3%	AEC	C-1	GC	83	3	1	8	4				DP	13	5	“
	Na_2_CO_3_ 3%	AEC	C-2	GC	72	11	5	7	4				DP	13	3	“
**Family Lessoniaceae**																
*Ecklonia cava*	HCl 0.1M hot	AEC	Ec-F1	HPLC	70		15	4		11			DP	19	ND	Ermakova et al. (2011)
	HCl 0.1M hot	AEC	Ec-F2	HPLC	57		16		23	4			DP	22	ND	“
	Enz.+CaCl_2_ 4M	PQA+AEC	F1	PAD	53	8	33		2	4			DP	20	16	Lee et al. (2012)
	Enz.+CaCl_2_ 4M	PQA+AEC	F2	PAD	60	4	31		1	4			DP	16	14	“
	Enz.+CaCl_2_ 4M	PQA+AEC	F3	PAD	78	8	10		2	2			DP	39	9	“
*Ecklonia kurome*	H_2_O+PQA	AEC+SEC	B-I	GC	34	34	13	18					DP	19	30	Nishino et al. (1989)
	H_2_O+PQA	AEC+SEC	C-I	GC	97		3						DP	47	2	“
	H_2_O+PQA	AEC+SEC	C-II	GC	83		17						DP	43	4	“
*Ecklonia maxima*	H_2_O hot	CaCl_2_ 1% +AP/R	EMF	HPLC	63	2	12	17	3	3			DP	21	tr.	Qu et al. (2014)
*Ecklonia radiata*	HCl pH 2 hot	CaCl_2_ 0.5%	6 min	HPLC	57		6		37				DP	22	2	Lorbeer et al. (2015)
	HCl pH 1 hot	Acetone ppt	AFS	HPLC	84	3	8	3	3				DP	28	1	Lorbeer et al. (2017)
*Eisenia bicyclis*	HCl 0.1M hot	AEC	EbF	HPLC	67	7	20	7					DP	14	ND	Ermakova et al. (2013)
*Lessonia nigrescens*	HCl pH 2 hot		B-Stipes	PC+GC	63	14	13	10					JL	6	29	Percival et al. (1983)
	HCl pH 2 hot		B-Frond	PC+GC	82	12		6					JL	7	17	“
	HCl pH 2+ Na_2_CO_3_ 3%	AEC	DF	PC+GC	57	13	21	9					JL	ND	ND	“
	H_2_O hot	CaCl_2_ 1% +AP/R	LNF	HPLC	65		11	14	4	6			DP	17	–	Qu et al. (2014)
*Lessonia trabeculata*	H_2_O hot	CaCl_2_ 1% +AP/R	LTF	HPLC	53	3	25	11	4	4			DP	16	tr.	Qu et al. (2014)
*Lessonia vadosa*	CaCl_2_ 2%+HCl 0.25M			GC	∼100	tr.	tr.						DP	38	–	Chandía and Matsuhiro (2008)
*Lessonia* sp.	CaCl_2_ 2% hot	AEC	B’-F1	GC	(~100	tr.	tr.						DP	37	4	Leal et al. (2018)

*^a^Key: AEC, anion exchange chromatography; SEC, size-exclusion chromatography; HC, hydrophobic chromatography; PQA, precipitation with quaternary ammonium salts; AP/R alcohol precipitation and redissolution; UF, ultrafiltration.*

*^b^Key for the less common abbreviations: PAD, HPAEC with pulse amperometric detector; GC, gas chromatography.*

*^c^Key: JL, method of Jones and Letham (1954); DP, method of Dodgson and Price (1962) or equivalent; IC, ion chromatography; EA, elemental analysis; Grav, gravimetric method.*

*^d^The information for the uronic acid is included in the molar ratio of monosaccharides.*

*^e^This family has been included recently in a separate order, the Chordales (Starko et al., 2019).*

Many galactofucans have been found within the Laminariaceae family, usually with low proportions of Xyl or Man. However, several fractions containing almost pure fucans have been found in *Laminaria angustata*, *L. hyperborea, Macrocystis pyrifera, Saccharina cichorioides*, and *S. japonica* (Table 5). For *L. angustata*, Nishino et al. (1994b) have isolated a homogalactan sulfate, probably in the only case that an almost fucose-free product is found within the “fucoidan” fractions of brown seaweeds. The trend showing mixtures of polysaccharides separable by charge also occurs for the products from the Laminariales: usually heterogeneous polymers, containing high proportions of uronic acids, and low sulfation appear in the early-eluting fractions of anion exchange chromatography, whereas highly sulfated fucans or galactofucans appear in the late-eluting fractions.

Seasonal differences were also observed: for *Costaria costata*, Imbs et al. (2009) determined that the proportion of Fuc, Gal, Glc, and sulfate increased from spring to summer, whereas those of Man, Rha, and Xyl decreased. This trend is similar to that observed by Men’shova et al. (2012) for *Padina pavonica* (see above). In another study, carried out for *Saccharina cichorioides* (as *Laminaria cichorioides*), it has been shown that after the summer, and through fall, the proportion of Fuc decreases again, whereas that of Man increases clearly (Anastyuk et al., 2010).

On the basis of chemical degradations and NMR spectroscopy, Bilan et al. (2010) arrived to many structural features of the fucoidans from *Saccharina lattisima*. Ehrig and Alban (2015) have shown the large effect of the marine habitat and season on the characteristics of the isolated fucoidans of this seaweed. Samples picked up in the Baltic Sea showed more laminaran contamination and lower fucoidan yields, fucose, and sulfate content than those collected around the Faroe Islands (regardless of the season), although the uronic acid content was similar. Regarding the season effects, the proportion of sulfate was higher in fucoidans from seaweeds collected in September than in May. Anion-exchange chromatography separation showed that only from the September-collected seaweed it was possible to obtain high yields of a high-fucose fraction with the highest biological activity. However, in a further work from the same group (Bittkau et al., 2020), the authors have isolated such a fraction with high fucose and sulfate content from the same North Atlantic location, in July without the need of any purification, suggesting that the year of collection has a major effect on the composition of the isolated fucoidans.

A study carried out with an unidentified species of *Alaria* (*Alaria* sp., Vishchuk et al., 2012) was later ascertained as being *A. ochotensis* (Prokofjeva et al., 2013). In the *Alaria* species studied so far, it is noteworthy to mention the presence of fucogalactans with approximately equal proportions of Fuc and Gal (Table 6).

For *Costaria costata*, high proportions of Man have been encountered in the polymers, especially in the less charged fractions isolated in some studies (Wang et al., 2014). In any case, Man appears conspicuously in most of the studies carried out on fucoidans of any origin.

The polysaccharides from *Undaria pinnatifida* were studied by many research groups, probably due to the fact that this seaweed, native from northeastern Asia, is very invasive and now is widespread all around the world (Casas et al., 2004; Thornber et al., 2004). It is worth noting that most of the studies have shown the presence of a galactofucan with high proportions of Gal, sometimes leveling out with Fuc. The proportion of other sugars (Man, Xyl and uronic acids) is usually low, whereas the proportion of sulfate is considerable, but lower than those of other species (Table 6).

### Other Orders

The analysis of the fucoidans of different species of the order Ectocarpales appears in Table 7. In this survey, only reports for ten different species (belonging to three families) of the order have been found. Highly sulfated galactofucans or homofucans coexist with polysaccharides containing significant proportions of Man, GlcA and/or Xyl.

**TABLE 7:** Reported compositions of the fucoidans from the orders Ascoseirales, Desmarestiales, Ectocarpales, Ralfsiales, and Scytothamnales.

Species	Extraction	Purification/	Acronym	Monosaccharide composition (moles %)									Sulfate		UA (%)	References
			Fractionation^a^		Method^b^	Fuc	Xyl	Gal	Man	Glc	Rha	GlcA	Others	Method^c^	%	
**Ascoseirales**																
*Ascoseira mirabilis*	CaCl_2_ 2% hot	AEC+SEC	1AF	PC+GC	29	9	19	9	10		25		JL	12	^d,e^	Finch et al. (1986)
	Na_2_CO_3_ 3% hot	AEC+SEC	3AF	PC+GC	17	9	31	14	9		17		JL	8	^d,e^	“
**Desmarestiales**																
*Desmarestia aculeata*	Na_2_CO_3_ 3% hot			GC+PC	21	3	41				35		JL	Low	^d^	Percival and Young (1974)
*Desmarestia firma*	H_2_O	AEC	F0.3M	GC+PC	X	X	X		∼50^f^		X	ManA X	JL	1	17	Carlberg et al. (1978)
*Desmarestia ligulata*	H_2_O	AEC	F0.2M	GC	52	3	5	1			38		JL	3	^d^	“
	H_2_O	AEC	F0.5M	GC	66	7	18	9					JL	20	4	“
*Desmarestia viridis*	HCl 0.1M hot	AEC+HC	DvF	GC	63	13	17	7				Ac	DP	12	ND	Shevchenko et al. (2017)
**Ectocarpales**																
**Family Adenocystaceae**																
*Adenocystis utricularis*	HCl pH 2 r.t.	PQA	EA1-5	GC	47	4	9	26	6	8			DP	5	42	Ponce et al. (2003)
	HCl pH 2 r.t.	PQA	EA1-20	GC	83		15	1					DP	23	4	“
	HCl pH 2 hot	PQA	EA2-5	GC	58	3	6	29	1	3			DP	6	31	“
	HCl pH 2 hot	PQA	EA2-20	GC	75	1	21	1	1	1			DP	21	6	“
**Family Chordariaceae**																
*Cladosiphon okamuranus*	HCl pH3	CaCl_2_ 3.5%+AEC		GC	86						14	Ac	DP	∼ 12	^d^	Nagaoka et al. (1999)
	ND			GC	91	2			7				DP	15	23	Cumashi et al. (2007)
	HCl 0.05M r.t.	CaCl_2_ 0.1M	CAF	PAD	99	1						Ac	DP	∼ 16	12	Teruya et al. (2009)
	ND	CE		GC	95	3	1						DP	15	9	Lim et al. (2019)
*Chordaria flagelliformis*	CaCl_2_ 2% hot	AEC	F2	GC	80	5	12		2			Ac	DP	18	16	Bilan et al. (2008)
	CaCl_2_ 2% hot	AEC	F3	GC	96		4					Ac	DP	27	13	“
	CaCl_2_ 2% hot	AEC	F4	GC	100							Ac	DP	27	10	“
*Dictyosiphon foeniculaceus*	CaCl_2_ 2% hot			GC	39	32	16	6	5				EA	9	10	Bittkau et al. (2020)
*Leathesia difformis*	HCl pH 2 r.t.		Ea	GC	90	6		4					DP	6	3	Feldman et al. (1999)
*Nemacystus decipiens*	H_2_O, Pressure		HN0	PAD	66	10	3	3	9			Fru 9,GalN 2	IC	20	36	Li et al. (2017)
	H_2_O	CaCl_2_ 3M+AEC	NP1	HPLC	74	3	5		2		15		DP	4	^d^	Cui et al. (2018)
	H_2_O+CaCl_2_	AEC+SEC	NP2	HPLC	76	2	2				20	Ac	DP	19	^d^	“
*Papenfussiella lutea*	H_2_SO_4_ 1% r.t.			GC	55	4	9	1	31					ND	5	Wozniak et al. (2015)
*Punctaria plantaginea*	CaCl_2_ 2% hot	PQA		GC	69	27	4						DP	19	2	Bilan et al. (2014)
**Family Scytosiphonaceae**																
*Chnoospora minima*	Enzymes pH 4.5 and 8	CaCl_2_+AEC	F2,1	PAD	19		38		7			NI^g^ 31, Ara 3	DP	5	ND	Fernando et al. (2017)
	Enzymes pH 4.5 and 8	CaCl_2_+AEC	F2,4	PAD	79		3					NI 18	DP	34	ND	“
	Enzymes pH 4.5	CaCl_2_ 5M	CMF	PAD	65	6	9		1			NI 19	DP	24	ND	Fernando et al. (2018)
*Scytosiphon lomentaria*	HCl pH 2 r.t.	PQA	A5	GC	38	15	15	24	3	5			DP	6	20	Ponce et al. (2019)
	HCl pH 2 r.t.	PQA	A30	GC	88		12						DP	29	2	“
**Ralfsiales**																	
*Analipus japonicus*	CaCl_2_ 2% hot	PQA+AEC	F_1_	GC	74	12	12	2				Ac	DP	13	12	Bilan et al. (2007)
	CaCl_2_ 2% hot	PQA+AEC	F_2_	GC	84	4	11					Ac	DP	23	6	“
**Scytothamnales**																
*Scytothamnus australis*	H_2_SO_4_ 1% r.t.			GC	92	3	2	1	2					ND	2	Wozniak et al. (2015)
*Splachnidium rugosum*	CaCl_2_ 2% hot			GC	86	7	3	2	2					ND	2	“

*^a^Key: AEC, anion exchange chromatography; SEC, size-exclusion chromatography; HC, hydrophobic chromatography; PQA, precipitation with quaternary ammonium salts; CE cation exchange.*

*^b^Key for the less common abbreviations: PAD, HPAEC with pulse amperometric detector; GC, gas chromatography.*

*^c^Key: JL, method of Jones and Letham (1954); DP, method of Dodgson and Price (1962) or equivalent; IC, ion chromatography; EA, elemental analysis.*

*^d^The information for the uronic acid is included in the molar ratio of monosaccharides.*

*^e^Even after purification, these samples contain 10–12% of alginic acid.*

*^f^Only the proportion of Glc is indicated. The remaining monosaccharides were not quantified.*

*^g^NI = sugar not identified.*

The analysis of the fucoidans from four species from the Desmarestiales is also shown in Table 7. It should be taken into account that these seaweeds contain free sulfuric acid in their vacuoles (Carlberg et al., 1978), making them very labile when taken out from the marine environment. This requires special techniques in order to obtain neutral extracts unaffected by the strong acid.

To the best of our knowledge, the fucoidans from only one species from the Ascoseirales and Ralfsiales, and two of the Scytothamnales have been studied (Table 7). The fucoidans from the three samples from the Ralfsiales and Scytothamnales appear to be particularly rich in Fuc and poor in uronic acids, whereas the *Ascoseira* sample was quite heterogeneous (Finch et al., 1986, Table 7).

## Concluding Remarks

The current review has surveyed most of the compositional data on fucoidans extracted from different species, in many cases after purification; more than 100 species were screened through the literature. Besides the obvious purpose of providing a reliable source of compositional data gathered in a set of tables, this review attempted to foresee if there is any correlation of these compositional data with their taxonomy, or if other factors are more important than the taxonomic origin.

These general considerations can be deduced from the analysis of the compositional data:

1. Separation by charge is the most efficient method to obtain “pure” fucoidan fractions. Either using anion-exchange chromatography with increasing concentrations of salt as eluant, or by precipitating with cationic detergents and redissolving at increasing ionic strengths, two main type of polymers can be separated: (a) those appearing at low ionic strengths, usually highly heterogeneous in their monosaccharidic composition (containing Fuc, Xyl, Gal, Man, Rha, GlcA), with low-sulfate content, and high uronic acid content, and b) those appearing at high ionic strengths, containing mainly Fuc, accompanied with variable proportions of Gal, highly sulfated and containing little (or none) uronic acids. Fractions containing intermediate proportions of both polysaccharides appear at medium ionic strengths. Figure 3 depicts the composition of fractions belonging to each of the first groups from selected seaweeds, showing clearly the marked differences between both groups. This behavior is observed for samples from the orders Fucales, Laminariales, Ascoseirales, Desmarestiales, Ectocarpales, and Ralfsiales (Mian and Percival, 1973; Carlberg et al., 1978; Bilan et al., 2002, 2013, 2016, 2018; Ponce et al., 2003, 2019; Ozawa et al., 2006; Mak et al., 2013); however, for the Dictyotales, the trend is obscured due to the abundance of Man and/or uronic acids in the products separated at each ionic strength (Table 4). It has been postulated that the biological activity is concentrated on the galactofucan components (Ponce et al., 2003, 2019; Croci et al., 2011).

**FIGURE 3:**
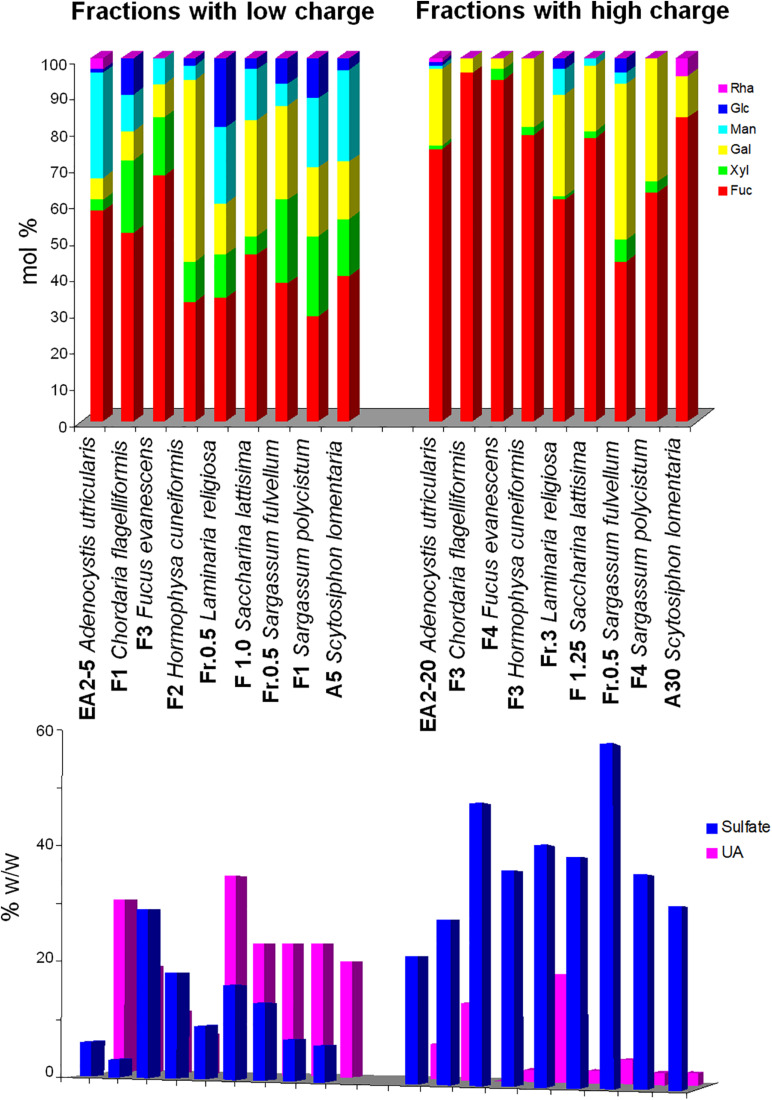
Difference in selected reported compositions of fucoidans submitted to charge-based separation methods. Fractions on the left side were eluted or redissolved at low ionic strengths, whereas those on the right side were eluted or redissolved at higher ionic strengths. Upper panel, neutral monosaccharide composition (mol/100 mols); lower panel, sulfate and uronic acid content. The data were reported by Koo et al. (2001), Bilan et al. (2002, 2008, 2010, 2013, 2018), and Ponce et al. (2003, 2019).
2. Acetate esters of the fucoidans are very common. As a matter of fact, this constituent has been found in almost every sample where it was searched. Determinations of acetyl groups are not very common, as they are only encountered through NMR spectra or specific colorimetric techniques. They are labile enough in mild alkaline or acid media as to get undetected when using some extraction procedures (Bernhard and Hammett, 1953; Wuts and Greene, 2006). Anyway, almost all of the seven tables report acetyl groups on some species. It is highly probable that searching in other species would have resulted in many more positive results.
3. In some cases, Man and Rha appear together, usually in fractions with lower sulfate contents. For Man, structural explanations have already been reported in terms of fucomannoglucuronans (Bilan et al., 2010), but for Rha no structural function has been found so far. Rha seems to appear in higher proportions within the order Dictyotales and the family Sargassaceae (Fucales).
4. The Dictyotales appear to be the most “atypical” order, as usually large proportions of Man and uronic acids appear. In one species which was highly fractionated, Man becomes the most important monosaccharide in the low-charged fractions, and it is still important in the fractions with more sulfate groups (Table 4; Rabanal et al., 2014). However, fractions with high proportions of monosaccharides different than Fuc were found in most of the taxa studied so far (see Tables).
5. The uronic acid content should be considered with due care. Sometimes it corresponds to GlcA actually comprising the fucoidan structure, but sometimes it corresponds to contamination with alginic acid (e.g., Finch et al., 1986; Lorbeer et al., 2017), a polysaccharide present in all of the brown seaweeds studied so far. By the same token, the Glc present in the samples should almost certainly correspond to contaminating laminarans (Lorbeer et al., 2017; Mateos-Aparicio et al., 2018). Only in a few cases, Glc has been shown to be part of the fucoidan structure (e.g., Duarte et al., 2001).
6. There are several factors to consider when comparing the compositional data of fucoidans from different seaweeds and research groups. The taxon is just one of them. Others like geographical location, year and season of harvest of the seaweed, extraction and purification methods, analytical methods, different parts or reproductive stages of the seaweeds are also of paramount importance in defining the final characteristics.
7. The geographic site of harvesting appears to be very important: Zvyagintseva et al. (2003) found marked differences between the fucoidans of *Fucus evanescens* collected in different spots of the southern Okhotsk Sea. Ehrig and Alban (2015) also found a significant difference between the composition and yields of fucoidans of *Saccharina lattisima* samples collected in the North Atlantic and in the Baltic Sea. This factor, together with the year of collection might explain the large differences in composition found for species studied by different groups (or at different times) even with similar extraction and purification procedures.
8. The season of harvesting has also influence over the composition of the fucoidans: a trend with increasing yields, and proportions of sulfate, Fuc, Gal and Glc (together with a decrease in the Man and Rha content) is observed as the collection month progressed from March to October, in the Northern Hemisphere (Imbs et al., 2009; Anastyuk et al., 2010; Men’shova et al., 2012; Ehrig and Alban, 2015).
9. The effect of the extraction conditions is more controversial: Ponce et al. (2003) and Wozniak et al. (2015) found very little differences when switching the extraction solvent from water to CaCl_2_ to diluted HCl. Alboofetileh et al. (2019b) found differences in yield and in sulfate content but a very similar monosaccharide composition using enzymes, ultrasound, or both combined. Rodríguez-Jasso et al. (2011) found a significant difference in composition and yields when changing the time and the pressure of a microwave-assisted water extraction. Nguyen et al. (2020) have shown a sharply different composition of the chemically and enzymatically-extracted crude products, being the latters richer in alginic acid and sulfate/Fuc ratios. After purification, the compositions might level off. However, the enzyme-aided extraction, also used by other groups (Dietrich et al., 1995; Albuquerque et al., 2004; Silva et al., 2005; Medeiros et al., 2008; Queiroz et al., 2008; Costa et al., 2011; Camara et al., 2011; Lee et al., 2012; Wang et al., 2014; Hu et al., 2016; Monsur et al., 2017; Fernando et al., 2017, 2018; Liu et al., 2018; Menezes et al., 2018; Song et al., 2018; Jayawardena et al., 2019; Alboofetileh et al., 2019a,b) appears to be an interesting prospect, considering cleaner chemical issues and the possibility of finding enhanced biological activities in comparison with chemically extracted products (Nguyen et al., 2020).

Some differences were found between the fucoidans isolated from reproductive and sterile tissue of five different seaweeds (Skriptsova et al., 2012, see Tables 1, 2, 5, 6). Usually the reproductive tissue is less heterogeneous, and carries more Fuc and less Glc than the sterile tissue. Regarding the extraction of fucoidans from different parts of the seaweeds, Percival et al. (1983) extracted separately the polysaccharides from fronds and stipes from *Lessonia nigrescens*, whereas Wozniak et al. (2015) compared the fucoidans isolated from reproductive structures and from vegetative structures in *Marginariella boryana*. The fucoidans from stipes and the vegetative structures, respectively, appear to be more heterogeneous (less Fuc and more uronic acids).

In order to obtain fucoidan samples devoid of contaminants, the best results were obtained by carrying out the extractions with dilute HCl or CaCl_2_, or using these agents after the extraction (for instance enzymatic) in order to precipitate the alginate in the first place, followed by a careful separation by charge (anion exchange chromatography eluting with increasing ionic strength, or precipitation with quaternary ammonium salts followed by redissolution with increasing ionic strengths). Further purification of each fraction by size-exclusion chromatography usually yield fucoidans devoid of alginic acid or laminaran contaminants.

The conclusion is that with so many variables determining the composition of the fucoidans, the subtle differences that might appear among the different higher taxa (order, family) surveyed in this review are overridden. Probably, comparisons carried out in the same labs with the same methods might help, or more profound structural studies might throw light on chemotaxonomical issues in the future.

## Author Contributions

NP was involved in the conceptualization, formal analysis, investigation, writing, and visualization of this work. CS was involved in the conceptualization, formal analysis, writing, visualization, and funding of this work. Both authors contributed to the article and approved the submitted version.

## Conflict of Interest

The authors declare that the research was conducted in the absence of any commercial or financial relationships that could be construed as a potential conflict of interest.
